# Modification of Gut Microbiota and Immune Responses via Dietary Protease in Soybean Meal-Based Protein Diets

**DOI:** 10.4014/jmb.2205.05033

**Published:** 2022-06-13

**Authors:** Minho Song, Byeonghyeon Kim, Jin Ho Cho, Hyunjin Kyoung, Jeehwan Choe, Jee-Yeon Cho, Younghoon Kim, Hyeun Bum Kim, Jeong Jae Lee

**Affiliations:** 1 Division of Animal and Dairy Science, Chungnam National University, Daejeon 34134, Republic of Korea; 2 Division of Food and Animal Science, Chungbuk National University, Cheongju 28644, Republic of Korea; 3 Department of Beef Science, Korea National College of Agriculture and Fisheries, Jeonju 54874, Republic of Korea; 4 DSM Nutrition Korea Ltd., Seoul 06675, Republic of Korea; 5 Department of Agricultural Biotechnology and Research Institute of Agriculture and Life Sciences, Seoul National University, Seoul 08826, Republic of Korea; 6 Department of Animal Resources Science, Dankook University, Cheonan 31116, Republic of Korea; 7 Institute of Agricultural Science and Technology, Kyungpook National University, Daegu 41566, Republic of Korea

**Keywords:** Diarrhea, dietary protease, gut microbiota, immune response

## Abstract

Plant-based protein sources such as soybean meal have low digestibility and are generally promoted accumulation of undigested proteins into the intestine by enzymatic treatments. Moreover, potential intestinal pathogens ferment undigested proteins, producing harmful substances, such as ammonia, amines and phenols, leading to an overactive immune response and diarrhea in weaned pigs. As a solution, dietary proteases hydrolyze soybean-based antinutritive factors, which negatively affect immune responses and gut microbiota. In this study, we investigated the effects of dietary proteases (PRO) in a low-crude protein (CP) commercial diet on the immune responses and gut microbiota of weaned pigs. The experimental design consisted of three dietary treatments: a commercial diet as a positive control (PC; phase1 CP = 23.71%; phase 2 CP: 22.36%), a lower CP diet than PC as negative control (NC; 0.61% less CP than PC), and NC diet supplement with 0.02% PRO. We found that PRO tended to decrease the frequency of diarrhea in the first two weeks after weaning compared with PC and NC. In addition, pigs fed PRO showed decreased TNF-α and TGF-β1 levels compared with those fed PC and NC. The PRO group had a higher relative proportion of the genus *Lactobacillus* and lower levels of the genus *Streptococcus* than the PC and NC groups. In conclusion, the addition of PRO to a low CP commercial weaned diet attenuated inflammatory responses and modified gut microbiota in weaned pigs.

## Introduction

Protein-utilizing microbes ferment undigested dietary protein, which induces diarrhea; the predominance of proteolytic bacteria damages intestinal epithelial cells through detrimental substances such as ammonia produced by fermentation [1]. Before weaning, the population of *Lactobacillus* is well established, but a decrease in its population promotes an increase in the proportion of potential pathogens. Establishing pathogenic microbiota adversely affects intestinal function by increasing the concentration of ammonia, amines, and phenols in the intestine and causing inflammation [2]. These harmful substances can alter intestinal morphology and induce diarrhea [3].

The addition of dietary protease (PRO) hydrolyzes soybean meal (SBM)-based anti-nutritional factors (ANFs), which may inhibit the production of pro-inflammatory cytokines during the digestive period [4-7]. These cytokines cause gut inflammation and induce further damage to the intestinal epithelium [8]. In particular, weaned pigs are vulnerable to diseases due to intestinal barrier breakdown because weaning stresses induce local inflammatory damage to epithelial cells [9]. Thus, the low activation of immune responses due to PRO addition can conserve energy and nutrients, thereby distributing it for growth and other functions in the weaned pigs [10].

Previous studies have reported that the use of a low-protein diet instead of antibiotics reduces excessive protein fermentation in the intestine and improves the health of piglets [11]. Moreover, the addition of exogenous proteases to swine diets increases protein utilization in the small intestine [12, 13]. Therefore, PRO addition to low-protein weaner diets was hypothesized to alter gut microbiota and other health parameters with potential benefits in weaned pigs. Thus, this study validates the beneficial effects of PRO addition to a commercial weaner diet with a reduced protein source on gut microbiota modulation and immune responses in weaned pigs.

## Materials and Methods

### Animals, Diets, and Study Design

All the animal experimental protocols were approved by the Institutional Animal Care and Use Committee of Chungnam National University, Daejeon, Korea (approval# CNU-00611). In total, 90 weaned pigs (Duroc × Landrace × Yorkshire) with an average body weight (BW) of 6.96 ± 0.06 kg and 28 days old were randomly assigned to three dietary treatments with 5 replicates of 6 pigs (3 barrows and 3 gilts) per pen using a randomized complete block design (block = BW). There is no difference in BW between the groups. The dietary treatments were 1) a commercial weaner diet to meet the requirement of crude protein (CP) as a positive control (PC; phase1 CP = 23.71%; phase2 CP = 22.36%), 2) a lower CP diet than PC as a negative control (NC; 0.61% less CP than PC), and 3) a NC diet supplemented with 0.02% dietary protease (PRO; 75,000 protease units/g). All dietary treatments were administered for 42 days. PRO is a commercial protease product (Ronozyme® ProAct, DSM nutrition products, Switzerland) from *Nocardiopsis prasina* expressed in *Bacillus licheniformis*. All dietary formulations met the nutritional requirements for weaned pigs based on the National Research Council [14] (Table S1).

### Sample Collection and Preparation for Analysis

The diarrhea incidence of each pig was visually checked daily from weaning to day 14, and the calculation of the diarrhea index was based on a previous report [15]. Feces for gut microbiota analysis were collected from three randomly selected pigs in each treatment group on the last day of the experiment and stored at −80°C until metagenomic analysis. Blood samples were collected from one randomly selected pig in each pen on days 1, 3, 7, and 14 post-weaning. Blood samples were collected through the jugular vein using EDTA tubes (Becton Dickinson Vacutainer Systems, Belliver Industrial Estate, Plymouth, PL6 7BP, UK) and serum tubes (Becton Dickinson Vacutainer Systems, USA) to yield whole blood and serum samples, respectively. The blood samples for serum separation were allowed to clot at room temperature for 1 h, and then centrifuged at 3,000 ×*g* for 15 min at 4°C. After centrifugation, the supernatant was collected and stored at −80°C until subsequent analysis.

### Inflammatory Response Analysis

Total white blood cell (WBC) counts in whole blood samples were analyzed using an automated hematology analyzer (SCIL Vet Animal Blood Counter; SCIL Animal Care Co., France) calibrated with porcine blood. The concentrations of cytokines and C-reactive protein (CRP) were measured using porcine ELISA kits according to the manufacturer’s instructions [tumor necrosis factor-α (TNF-α; Genorise Scientific, Inc., USA), transforming growth factor-β1 (TGF-β1; Genorise Scientific, Inc.), and CRP (Genorise Scientific, Inc.). All cytokine measurements were performed as previously described [16, 17]. Data were determined using a microplate reader at 450 nm (Epoch microplate spectrophotometer; BioTek Instruments Inc., USA).

### 16S rRNA Gene Sequencing and Gut Microbiota Analysis

The total DNA of each fecal sample (300 mg) was extracted using the QIAamp DNA Stool Mini Kit (Qiagen, Germany), according to the manufacturer’s protocol. Genomic DNA was assessed for quality and concentration using a NanoDrop ND-1000 spectrophotometer (NanoDrop Technologies, USA) and then stored for analysis at -80°C. The V4 region of the 16S rRNA gene was amplified by the polymerase chain reaction (PCR) using featured primers as listed previously [18]. Amplicons were sequenced using the Illumina MiSeq platform according to the manufacturer's protocol. All sequencing was conducted by Macrogen Inc. (Korea). Raw sequence data were analyzed using mothur software, and low-quality sequences were removed [19]. Sequencing errors and chimeras were eliminated using the UCHIME algorithm implemented in the Mothur processing [18]. The remaining high-quality sequences were categorized into operational taxonomic unit (OTUs) clustering according to an identity-cutoff of ≥ 97% [20]. The sequence number was normalized by random subsampling for the downstream analyses of microbial alpha diversity, such as phylogenetic information, observed OTUs, Chao1, Shannon, and Simpson indices, and beta diversity (PCoA; principal coordinate analysis).

### Statistical Analysis

Data were analyzed using the general linear model procedure of SAS (Version 9.4, 2013, SAS Inc., USA) in a randomized complete block design, with initial BW as a block. A pen was an experimental unit. The statistical model for cytokine and CRP concentrations and WBC counts included the effects of dietary treatment as a fixed effect. The chi-squared test was used to analyze the frequency of diarrhea. Alpha diversity, taxonomic classification and beta diversity of microbial populations among dietary treatments were analyzed using Prism software (Prism 5.00; GraphPad Software, USA) and MicrobiomeAnalyst (https://www.microbiomeanalyst.ca/), respectively. Statistical significance and tendency were considered at *p* < 0.05 and 0.05 ≤ *p* < 0.10, respectively.

## Results

### Diarrhea Incidence and Inflammatory Responses

**Fig. 1:**
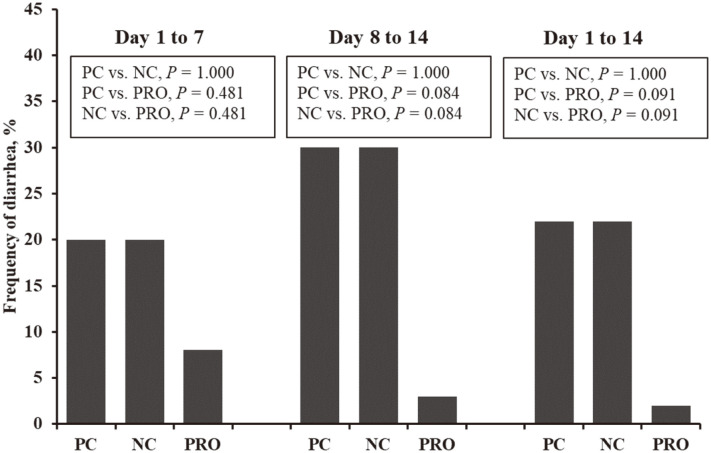
Diarrhea frequency of weaned pigs fed positive control (PC), negative control (NC), and NC + 0.02% dietary protease supplementation (PRO) diets for the first two weeks after weaning. Each bar represents the frequency of diarrhea (%, the number of diarrhea/number of pen days × 100). The data were analyzed using the chi-squared test.

**Fig. 2:**
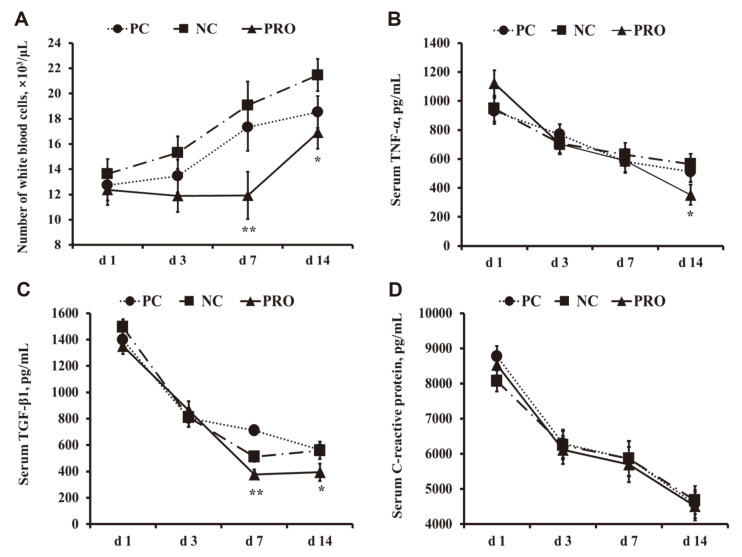
Number of white blood cells (**A**), serum TNF-α (**B**), serum TGF-β1 (C), and serum C-reactive protein (D) in weaned pigs fed positive control (PC), negative control (NC), and NC + 0.02% dietary protease supplementation (PRO) diets. Each value is the mean of five replicates. The *p* < 0.05 and 0.05 ≤ *p* < 0.10 were indicated as ** and *, respectively.

Pigs fed PRO tended to decrease (*p* < 0.10) the frequency of diarrhea from days 1 to 14 after weaning compared with those fed PC and NC (Fig. 1). In addition, pigs fed PRO had reduced WBC numbers on days 7 (*p* < 0.05) and 14 (*p* < 0.10) (Fig. 2A) after weaning compared with those fed PC and NC. Furthermore, dietary PRO and PC tended to have lower (*p* < 0.10) WBC numbers and TNF-α levels on day 14 than NC (Figs. 2A and 2B). Pigs fed PRO showed decreased (*p* < 0.05) TGF-β1 on day 7 compared with those fed PC and NC (Fig. 2C). However, no differences were observed in the CRP levels among the dietary treatments (Fig. 2D).

### Diversity and Classification of Gut Microbiota

**Fig. 3:**
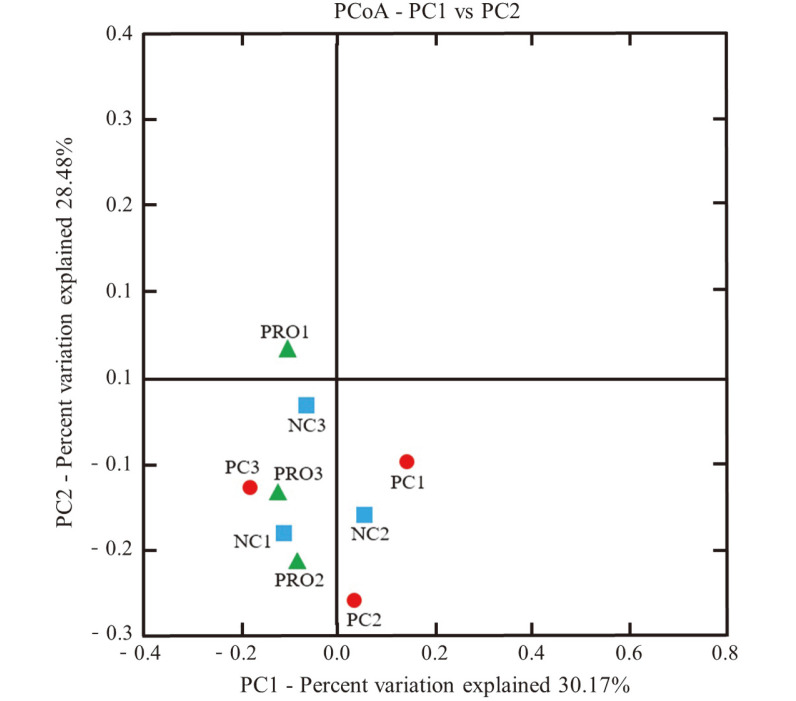
Discriminant analysis of principal components among fecal samples at day 42. The nine differentially abundant bacterial genera represent the number of variables in the model. Individual pig samples with treatments are designated with the following symbols: PC (red, ○): positive control; NC (blue, □): negative control; PRO (green, △): NC + 0.02% dietary protease.

**Fig. 4:**
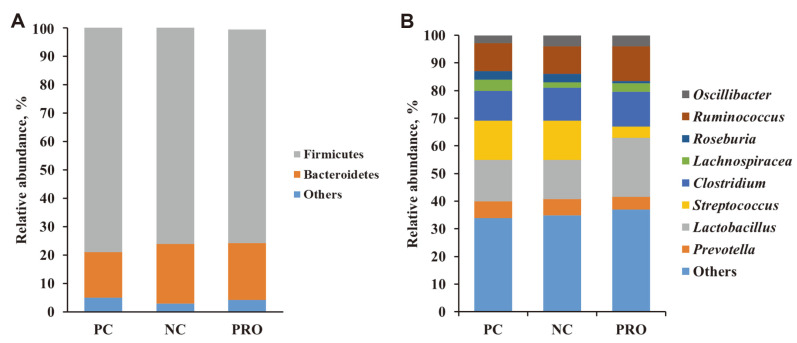
Taxonomic classification of total bacteria at the phylum level (**A**) and genus level (**B**) retrieved from pooled DNA amplicons from feces of PC, NC, and PRO pigs (*n* = 3; day 42). PC: positive control; NC: negative control; PRO: NC + 0.02% dietary protease.

Fecal samples were analyzed for alpha diversity using high-throughput sequencing. Samples were collected from the PC, NC, and PRO treatment groups, and the average bacterial sequencing numbers were 11,888, 9,867, and 11,608, respectively (Table 1). The diversity richness (OTUs), Chao 1, Shannon, and Simpson indices did not differ among the PC, NC, and PRO treatment groups (Table 1). In addition, beta diversity in each dietary treatment group was examined using UniFrac PCoA. However, the PCoA results showed no distinction in the discriminant analysis among treatments (Fig. 3). The 16S rRNA sequencing reads were classified into different taxa and the relative abundance of each sample was determined at the phylum and genus levels (Fig. 4). At the phylum level, Firmicutes and Bacteroidetes were dominant phyla, representing approximately 95% of the total sequences (Fig. 4A). At the genus level, PRO supplementation increased the proportion of *Lactobacillus* and decreased the population of *Streptococcus* significantly compared with PC and NC (Fig. 4B).

## Discussion

Weaned pigs spend more energy to increase the function of the gastrointestinal tract and to combat external sources of infection rather than digesting and absorbing nutrients to adapt to the changed environment. Moreover, diarrhea, which occurs during the post-weaning period, accelerates apoptosis of intestinal mucosal cells and restricts regeneration, thereby leading to growth inhibition in weaned pigs. Dietary protease addition overcomes these nutritional disadvantages by decreasing the apparent ileal digestibility of CP and positively affects the growth performance of weaned pigs. Improved protein digestion and absorption by PRO reduce the flow of undigested proteins into the large intestine, thereby preventing the proliferation of pathogenic microbes and their harmful metabolites [21].

Bacterial metabolites or toxins produced by SBM-based ANFs in the gut cause inflammation [22], which is accompanied by damage to epithelial cells and a decrease in growth efficiency [23, 24]. However, PRO addition prevented inflammation of gut epithelial cells by degrading the feed antigen in SBM, which may lead to decrease diarrhea [25]. This finding is in agreement with the diarrhea frequency results of the present study.

Neonatal pigs maintain their immune systems by depending on passive immunity through colostrum antibody absorption before developing active immunity, starting around three weeks after birth [26]. However, premature weaning compromises the undeveloped gastrointestinal barrier function caused by weaning stressors and increases intestinal epithelial permeability [9]. As a result, weaned pigs are vulnerable to infection that is directly related to growth retardation. Moreover, immediate conversion to a solid diet causes gut epithelial inflammation [9]. In particular, soybeans contain allergenic substances that cause villous atrophy, decrease growth performance, and impair intestinal integrity [27, 28]. The results of the present study also confirmed that PRO addition can reduce the number of WBC, an indicator of inflammatory responses. This is in agreement with previous studies [6, 29], which reported that PRO addition in corn- and SBM-based diets improved growth performance by enhancing the degradation of the protein-disulfide bond of ANFs. This feed antigen can cause local inflammation in the intestine and increase the production of pro-inflammatory cytokines, such as TNF-α, interleukin-1, and interleukin-6 [30]. Furthermore, these inflammatory reactions have an adverse effect on epithelial cell differentiation. Intestinal morphology (villus atrophy and crypt hyperplasia) and function are affected along with abrupt biological and morphological changes in the intestine caused by weaning, further inducing growth deterioration [23, 30].

In the current study, PRO addition suppressed the secretion of serum TNF-α and TGF-β1. This observation is consistent with that of a previous study showing decreased pro-inflammatory cytokine production due to proteolytic enzymes [6]. In addition, pro-inflammatory cytokines, such as TNF-α, negatively affect the permeability of intestinal epithelial tight junctions [25], and TGF-β1 is classified as an anti- or pro-inflammatory cytokine under various conditions [6]. These results indicate that PRO may contribute to reducing systemic inflammation in weaned pigs, and this retained energy and nutrients may contribute to growth.

Intestinal bacterial flora changes after weaning with an increase in both the complexity and stability of the microbial community [1, 25, 31, 32]. During this period, inadequate colonization of microorganisms results in an imbalance between commensal bacteria and pathogens [24]. The commensal bacteria (non-pathogenic) have the following essential roles: 1) protection by forming a barrier against the pathogen, 2) aid in digestion and metabolism, such as vitamin synthesis, and 3) promote growth and differentiation of epithelial cells [25, 33]. Therefore, adequate microbial colonization and, eubiosis are essential for subsequent growth and health of weaned pigs.

The microbiota of neonatal pigs is primarily affected by the maternal microbiota after birth, however, that of weaned pigs is influenced by the feed [32, 34, 35]. The feed intake of the weaned pigs was generally low after weaning. Anorexia induces a compensatory pattern during the first week after weaning, causing an increase in the flow of undigested proteins into the large intestine [36]. However, most undigested proteins are fermented in the large intestine by harmful microbes, such as *Clostridium* and several other pathogens, releasing nitrogenous compounds [1, 2, 37]. Therefore, an appropriate feeding strategy is required to enhance the colonization of commensal species (*i.e.*, *Lactobacillus*) post-weaning and establish a balance between commensal microbiota and pathogenic organisms. These would aid in enhanced protein digestibility and absorption in the small intestine to prevent the influx of undigested substrates into the lower part of the intestine and fermentation in the large intestine.

In the present study, PRO addition induced an increase in the relative proportion of *Lactobacillus* compared with that in PC and NC treatments at the genus level. A previous study also reported a similar increase in the counts of *Lactobacillus* after dietary supplementation with multiple-enzymes and a decrease in the populations of *Salmonella* and *Escherichia coli* in feces [31]. Although we observed no change in the population of *E. coli*, the population of *Streptococcus* was decreased on PRO supplementation. Streptococci are common in all animals, and several streptococcal species are not pathogenic. However, the major species in the pig is *S. suis* that causes septicemia, meningitis, endocarditis, arthritis, and other infections [38]. It was anticipated that the addition of PRO to the diet would cause gut microbiota modulation by increasing the amount of probiotics, such as *Lactobacillus*. Studies have reported that a mono-component protease or an enzyme cocktail containing PRO can improve the growth, nutrient utilization, and intestinal health of pigs [4, 5, 12, 31]. A common finding across these studies was that the population of beneficial bacteria increased, whereas that of harmful bacteria decreased after exogenous proteolytic enzyme supplementation.

The results of this study support that the immune response and diarrhea frequency decreased due to a positive change in the intestinal environment with the addition of PRO to low-crude protein commercial weaner diets. This led to increase in the beneficial gut microbiota, such as *Lactobacillus* spp. Therefore, PRO is a promising way to reduce protein requirements in swine diets, with gut health benefits.

## Supplemental Materials

Supplementary data for this paper are available on-line only at http://jmb.or.kr.

## Figures and Tables

**Table 1:** Number of sequences, observed diversity richness (OTUs), and diversity estimates of bacteria in feces^1^.

Items	Dietary treatment			SEM	*P*-value

	PC	NC	PRO		
No. of Seq.	11,887.67	9,867.00	11,608.33	1336.49	0.546
OTUs	331.33	300.33	326.00	19.07	0.510
Chao1	360.68	344.32	362.08	19.05	0.773
Shannon	5.84	5.73	5.94	0.29	0.880
Simpson	0.94	0.95	0.95	0.01	0.740

^1^Each value is the mean of 3 replicates per treatment.

PC, positive control; NC, negative control; PRO, NC + 0.02% dietary protease; SEM, standard error of mean; OTUs, operational taxonomic units.
