# Supplementary material for: Modification of Gut Microbiota and Immune Responses via Dietary Protease in Soybean Meal-Based Protein Diets
Source: J Microbiol Biotechnol. 2022 Jun 13;32(7):885–91. doi: 10.4014/jmb.2205.05033 (PMC9628920; doi:10.4014/jmb.2205.05033)
Supplement: Supplementary file 1 [file jmb-32-7-885-supple.pdf]

J. Microbiol. Biotechnol.
https://doi.org/10.4014/jmb.2205.05033

Supplementary Tables

Table 1. Composition of the commercial diets for weaned pigs (as-fed basis)[1)]

Items	Phase 1		Phase 2	
	PC	NC	PC	NC
**Ingredients, %**				
Corn	45.00	47.00	50.86	52.58
Soybean meal (44%)	18.00	16.00	27.00	25.28
Dried whey	15.00	15.00	10.00	10.00
Soy protein concentrate	-	-	-	-
Fish meal	11.46	11.46	8.00	8.00
Spray-dried plasma	4.00	4.00	-	-
Lactose	3.00	3.00	-	-
Soybean oil	3.00	3.00	3.00	3.00
Limestone	0.50	0.50	0.60	0.60
Monocalcium phosphate	-	-	0.40	0.40
Vit-Min premix[2)]	0.04	0.04	0.04	0.04
L-lysine-HCl	-	-	0.10	0.10
DL-methionine	-	-	-	-
L-threonine	-	-	-	-
Total	100.00	100.00	100.00	100.00
**Calculated nutrient compositions**				
Metabolizable energy, Mcal/kg	3.54	3.54	3.49	3.49
Crude protein, %	23.71	23.10	22.36	21.75

Calcium, %	0.85	0.85	0.82	0.82
Phosphorus, %	0.72	0.72	0.68	0.68
Lysine, %	1.54	1.50	1.40	1.36
Methionine, %	0.44	0.43	0.41	0.40
Threonine, %	1.02	0.97	0.90	0.85
Tryptophan, %	0.29	0.29	0.26	0.26
Cysteine, %	0.42	-	0.35	-
Arginine, %	1.39	1.36	1.38	1.35
Histidine, %	0.63	0.62	0.60	0.59
Isoleucine, %	0.98	0.96	0.94	0.92
Leucine, %	2.02	1.99	1.88	1.85
Phenylalanine, %	1.09	-	1.04	-
Valine, %	1.17	1.14	1.02	0.99

[1)]Phase 1: week 1 to 3 (21 days); phase 2: week 4 to 6 (21 days); PC, positive control; NC, negative control.

[2)]Provided per kilogram of diet: vitamin A, 12,000 IU; vitamin D_3, 2,500 IU; vitamin E, 30 IU; vitamin K_3, 3 mg; D-pantothenic acid, 15 mg; nicotinic acid, 40 mg; choline, 400 mg; and vitamin B_{12}, 12 μg; Fe, 90 mg from iron sulfate; Cu, 8.8 mg from copper sulfate; Zn, 100 mg from zinc oxide; Mn, 54 mg from manganese oxide; I, 0.35 mg from potassium iodide; Se, 0.30 mg from sodium selenite.
